# Hybrid Mesoporous Bifunctional Adsorbents of Pb^II^ and Cd^II^ Ions from Aqueous Solution at Ambient Temperature

**DOI:** 10.1002/open.202500565

**Published:** 2026-04-28

**Authors:** Rokia Bouri, Soumia Chirani, Fazilet Guechairi, Leila Cherif‐Aouali, Karima Saidi‐Bendahou, Renaud Denoyel

**Affiliations:** ^1^ Département de Chimie Institution Université Abou Bekr Belkaid Tlemcen Algerie; ^2^ Département de Pharmacie Institution Université Djillali Liabbes Sidi Bel Abbés Algerie; ^3^ Département de Chimie Institution Aix‐Marseille Université Marseille France

**Keywords:** adsorption, bifunctional adsorbents, grafting, heavy metals

## Abstract

In the current work, the possibility of introducing simultaneously two different functions onto the surface of mesoporous material SBA‐15 was achieved. In fact, the thiol and amino organic functions, respectively, were incorporated according to post and successive grafting. The rate of grafting of these ligands was discussed using the following characterization methods: XRD, N_2_ adsorption–desorption, and FT‐IR. A dramatic decrease was found in the textural characteristics due to the success grafting also evidenced by FT‐IR spectroscopy and XRD analyses. After that, the adsorption study of Pb^2+^ and Cd^2+^ from aqueous solution using the resulting bifunctional adsorbents at ambient temperature shows significant exaltation for lead adsorbed quantities against reduction of cadmium adsorbed ones; this behavior is due to the synergic effect coming from the simultaneous existence of –SH and –NH_2_ fragments. Thus, the adsorption of Pb^2+^ on both adsorbents is more efficient (medium adsorption rate = 84%) compared to Cd^2+^ (adsorption rate does not exceed 10%). The maximum adsorption capacities are found between 164 and 168 mg/g for lead ions and between 19 and 16 mg/g for cadmium ions. The kinetic study demonstrates that the process is moderately fast; the equilibrium is achieved within 50 min, whereas the kinetics analysis revealed that the overall Pb^2+^ and Cd^2+^ adsorption process by two adsorbents was more consistent with the pseudo‐second‐order model. Besides, the adsorption isotherms fit well the Freundlich model. Additionally, high adsorption efficiencies were attained at the pH of each metal solution, around 5.3 for lead and 6.1 for cadmium. Moreover, the reuse performance shows the possibility of efficiently reusing these adsorbents even after three cycles, which means that the synergic effect is also maintained.

## Graphical abstract

The bifunctionalization of the surface of mesoporous material SBA‐15 by thiol and amino as two organic functions via post‐ or successive grafting leads to obtaining a high performance and effective adsorbent for lead ion removal from aqueous solution. In contrast, there was a reduction in the cadmium ion removal rate. The simultaneous incorporation of –SH and –NH_2_ fragments generates a characteristic synergic effect of the resulted materials.

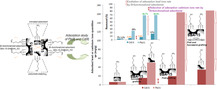

## 1 Introduction

The contamination of water and wastewater by different pollutants, such as heavy metals due to the accelerating pace of industrialization results in extensive damage to the ecosystems [1, 2]. In general, heavy metals are not biodegradable and are well recognized by their high toxicity [3, 4], their high solubility because they are easily absorbed by aquatic and soil species, which increases their concentration and accumulation [5] along tropical chains [6]. The removal of heavy metals was realized by numerous techniques, including, ion exchange, precipitation, coagulation, membrane, adsorption, phytofiltration, complexation, reverse osmosis, and electrochemical methods [7, 8, 9, 10, 11, 12, 13, 14]. Due to the low cost of adsorbents, high efficiency for low‐charged heavy metals wastewater treatment, effective, economic, and simple design and good selectivity, adsorption has received much attention for heavy metal remediation, spatially when in the form of free ions [15, 16].

The permissible concentrations of lead (Pb) and cadmium (Cd) in drinking water are 0.01 and 0.005 ppm, respectively, according to the World Health Organization (WHO) and the United States Environmental Protection Agency (USEPA) [17]. Furthermore, exceeding these limits can cause serious harm to human health, for example, exposure to lead provokes neurological damage [18] and cadmium is a known carcinogen [5].

Since their discovery, the highly ordered mesoporous material SBA‐15 with a regular two dimensional hexagonal array of channels has been the main subject of an indeterminable number of scientific researches [19]. The functionalization of SBA‐15 by thiol and amino‐based groups (thiourea, thioether, diamine, triamine, etc.) was widely reported in the literature and their use as heavy metal remediation adsorbents has been extensively studied [20, 21, 22, 23]. Therefore, there are two main approaches to chemically bind functional groups, postsynthesis or cocondensation (direct synthesis) [24]. Consequently, the resulting hybrid materials will have different terminal groups as active sites of heavy metal complexations. According to Liang et al. [25], thiol functionalized mesoporous SBA‐15 material was showed special affinity for lead ions and moderately for cadmium ions. The high affinity of sulfur‐based ligands such as Mercaptopropyltrimethoxysilane (thiol organic function) is justified according to Person's hard–soft, acid base (HSAB) theory [26, 27]. While equal adsorption capacity to Pb^2+^ and Cd^2+^ ions from wastewater was obtained using multiamine functionalized mesoporous silica SBA‐15 as adsorbents [28]. In general, the functional groups are defined by their local hydrophobic/hydrophilic environment, which is the origin of the activity of functionalized materials [29]. Furthermore, the incorporation of two functional groups on the same mesoporous material, knowing as bifunctionalization, allows obtaining of mesoporous hybrid materials with desirable properties. It can release it by adjusting the type and ratio of the functional groups [30]. In addition, the bifunctionnallizing technology can enhance the retention of varied pollutants as it has been reported in the literature [31, 32, 33, 34].

**SCHEME 1:**
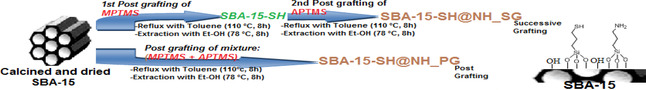
Schematic illustration of synthesis of SBA‐15‐SH@NH bifunctionalized materials.

In this work, the simultaneous grafting of 3‐mercaptopropyltriethoxysilane (MPTES) and 3‐aminopropyltriethoxysilane (APTES) onto the surface of SBA‐15 mesoporous material was investigated respectively through successive grafting (SG) and postgrafting (PG) as shown in Scheme 1. The adsorption at ambient temperature of lead and cadmium ions using the resulting adsorbents was reached by the study of the influence of key parameters such as contact time, initial metal concentration, and pH. The results of the reuse of adsorbents were also presented.

The current study aims to design bifunctionalized materials for the removal of Pb^2+^ and Cd^2+^ from aqueous solution. For this, a comparative study between the retention capacities of Pb^2+^ and Cd^2+^ using mono and bifunctionalized adsorbents is established and enriched by a detailed discussion together of the kinetics and mechanism of adsorption.

## 2 Results and Discussion

### 2.1 Characterization Results

#### 2.1.1 Low‐angle X‐ray Diffraction

**FIGURE 1:**
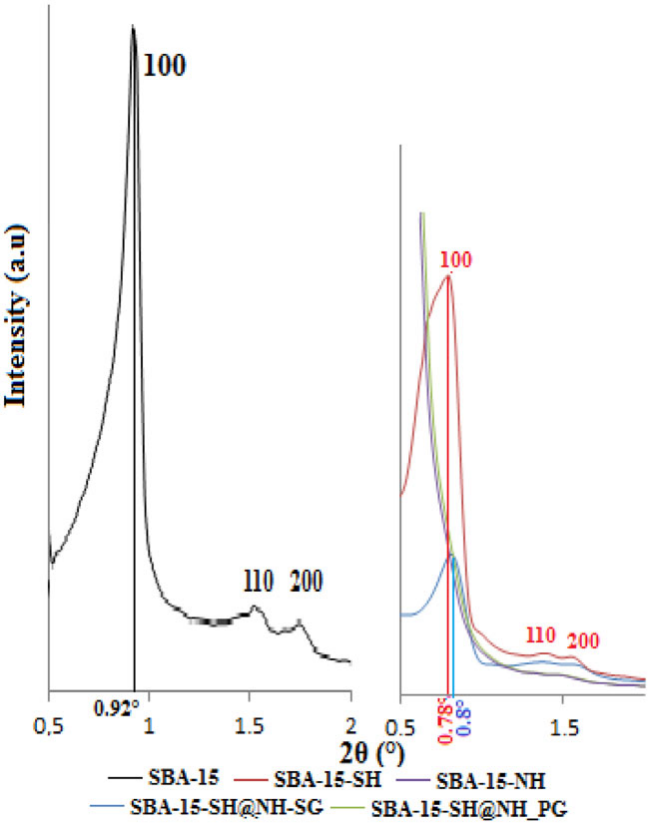
Powder XRD patterns for calcined SBA‐15, SBA‐15‐SH, SBA‐15‐NH, SBA‐15‐SH@NH_SG, and SBA‐15‐SH@NH_PG samples.

The XRD patterns of different samples before and after modification with MPTES and APTES are shown in Figure 1. For the calcined SBA‐15 silica, the XRD diffractogram exhibits three well‐conventional diffraction peaks at low 2*θ*, one very sharp (100) diffraction peak at 0.92° and two weak peaks (110 and 200) at 1.52° and 1.76°, respectively, associated with *p6mm* hexagonal symmetry [35]. After modification with MPTES, the XRD patterns of SBA‐15‐SH show the presence of the same peaks as SBA‐15 silica, with a lower intensity of the peak (100). In contrast, no peak was observed for the monofunctionalized sample SBA‐15‐NH. In the case of bifunctional samples, the three peaks were obtained with very low intensities in the SBA‐15‐SH@NH_SG pattern, but the SBA‐15‐SH@NH_PG diffractogram shows no peak.

The decrease in the (100) reflection strength of functionalized samples (SBA‐15‐SH and SBA‐15‐SH@NH_SG) can be explained by the scattering contrast dependence between the pore channels and silica walls after binding of organic groups to the pore surface silica [36, 37]. The high concentration of MPTMS, when preparing the monothiolated sample, had direct effects on the formation of P123 micelles, and on the interaction between P123 surfactant and silicates (TEOS) [38]. This causes a disturbance in the mesostructured formation [39] which becomes reinforced with the addition of amine functions. All these observations reflect further evidence of functionalization occurring mainly inside the mesopore channels [20]. On the contrary, for the two other functionalized samples (SBA‐15‐NH and SBA‐15‐SH@NH_PG), no peak was observed because the presence of amino groups NH_2_ and silanol SiOH can lead to hydrogen bond formation, constituting a cyclic structure which takes up more spaces in the mesoporous structure [40]. Generally, the disappearance of the intense (100) peak is noticed with the postsynthesis procedure, when cocondensation route reassures its presence, whatever the concentration of the organosilane [30].

#### 2.1.2 Nitrogen Adsorption–Desorption Isotherm

**FIGURE 2:**
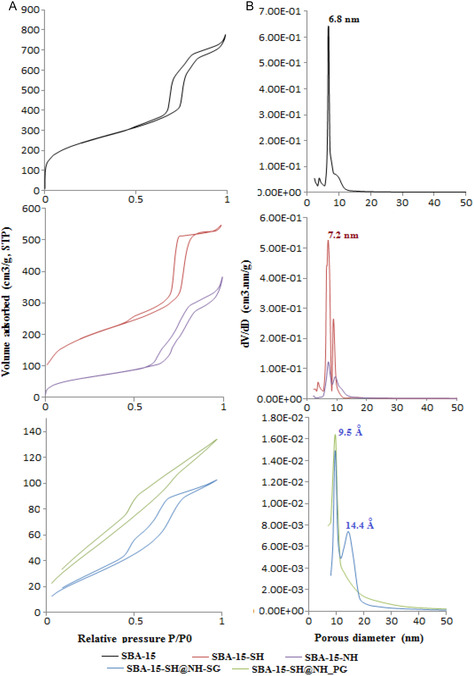
N_2_ adsorption–desorption isotherms of prepared samples (Graphs A) and their corresponding porous distribution (Graphs B).

Figure 2A depicts N_2_ adsorption–desorption isotherms for all samples. For SBA‐15, SBA‐15‐SH, and SBA‐15‐NH samples, a typical irreversible type IV curve in the BET categorization system [41] with an H1 hysteresis loop type is obtained, indicative of the existence of micropores and mesopores with the capillary condensation phenomenon. However, the inflection position shifted slightly toward a lower relative pressure of the prepared bifunctionalized samples; the desorption branches of their isotherms show a close hysteresis at relative pressure from 0.2 to 0.4, approximately with a gradual decrease in their pore diameters toward micropore ranges (see Figure 2B). Indeed, the same result was announced by Kao et al. [42].

Structural and textural properties of all samples are summarized in Table 1. As observed, the textural characteristics of functionalized samples were diminished after modification of the SBA‐15 surface, which was extensively reported in the literature [20, 25, 28]. However, this diminution was more pronounced for bifunctionalized samples because the adsorption of N_2_ molecules is partially blocked due to the large volume of the two organic functions. At this time, the incorporation rates of both thiol and amino groups by postgrafting of the mixture (MPTMS + APTMS) are higher than that of SG, which further explains the reduction in surface area of one compared to the other. On the other hand, the bifunctionalized materials have similar pore size values, suggesting that the post or SG has no effect on their pore structure.

**TABLE 1:** Structural and textural properties of SBA‐15; SBA‐15‐SH; SBA‐15‐NH; SBA‐15‐SH@NH_SG, and SBA‐15‐SH@NH_PS samples.

Samples	*S*_BET_ a, m^2^/g	Vp^b^, cm^3^/g	Dp^c^, nm	2*θ,* °	*d*_100_ d, (Å)	*a* e, Å
SBA‐15	824	1.04	6.8	0.92	9.46	11.1
SBA‐15‐SH	665_**(25%)**_ f	0.51	9.1	0.8	11.04	12.7
SBA‐15‐NH	222_**(45%)**_ f	0.55	7.9	—	—	—
SBA‐15‐SH@NH_SG	177_**(17%;15%)**_ f	0.16	0.95	0.78	11.32	13.1
SBA‐15‐SH@NH_PG	105_**(21%;23%)**_ f	0.15	0.95	—	—	—

a Specific surface area determined by BET method.

b Total pore volume.

c Mean pore size calculated from desorption branch of isotherm by BJH method.

d *d*_100_ = 2.sin*θ*/n. *λ*_cu_ (*n* = 1 and *λ*_cu_ = 1. 54 178 Å).

e *a* = 2. d_100_/√3.

f The incorporation rate of thiol, amino or both groups.

Consequently, this decrease of textural characteristics for the resulting functionalized materials indicates the successful coupling of the organic silane on the pore surface of SBA‐15 [28, 43].

#### 2.1.3 FT‐IR Study

**FIGURE 3:**
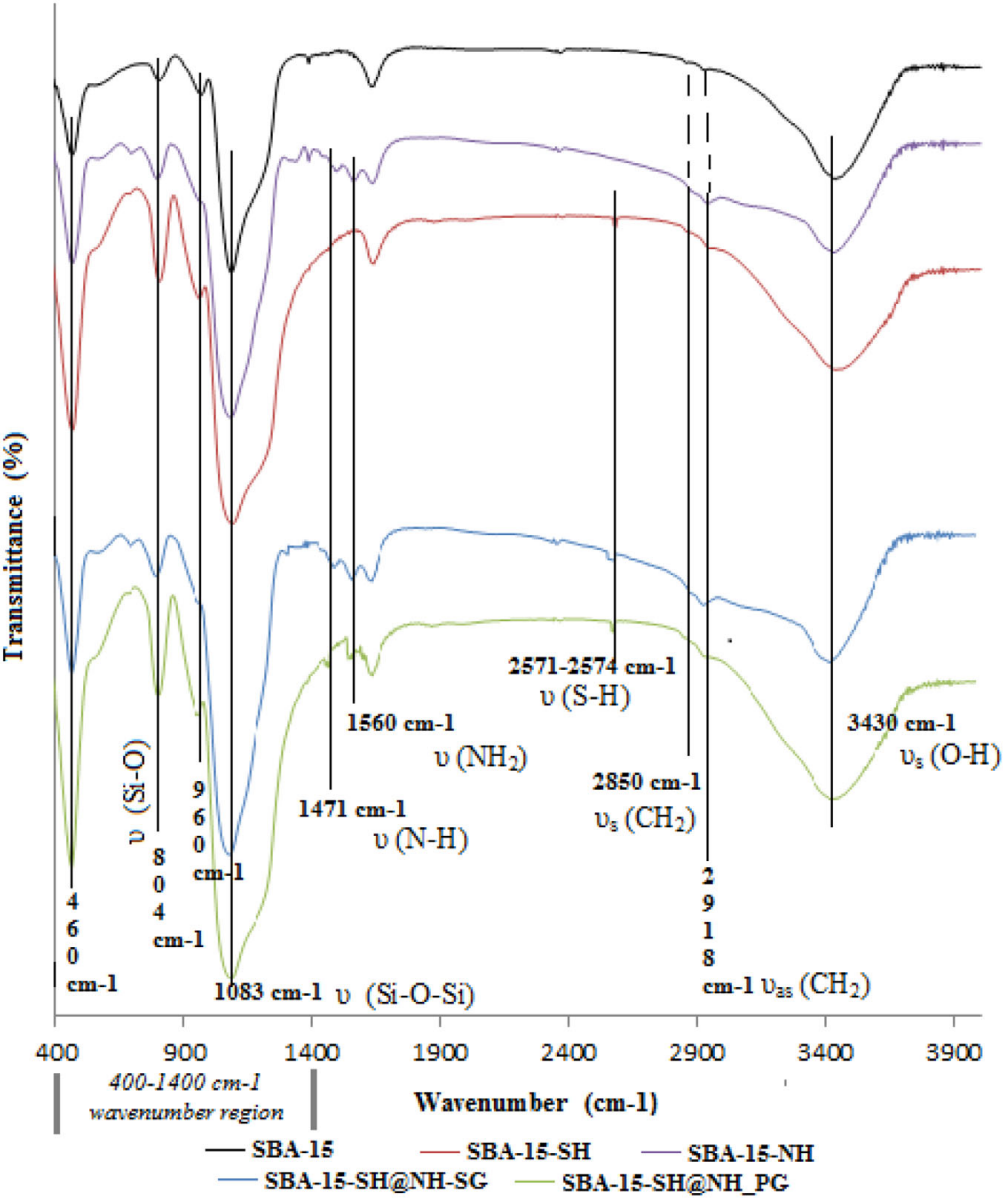
FT‐IR spectrum of different samples.

For all functionalized samples, the IR spectrum in the wavenumber region between 400 and 1400 cm^−1^ presents the same characteristic bands of the silica framework of the SBA‐15 material (see Figure 3) [44]. For samples containing mercaptopropyl groups, a distinctive absorption band of the thiol groups appears at around 2571–2574 cm^−1^, corresponding to the S—H band vibration [20, 25]. In the case of samples that have aminopropyl groups, the two broad bands seen at 1560 and 1471 cm^−1^ correspond to NH_2_ and N—H deformations, respectively [21, 28]. The spectrum shows again two other peaks at 2850 and 2918 cm^−1^ attributed to C—H band vibration from mercaptopropyl and aminopropyl groups; meanwhile, the C—H band vibration still exists in the SBA‐15 spectrum due to the residual presence of triblock copolymer (P123). The Si—OH vibration band was also seen at 3430 cm^−1^ [45].

## 3 Adsorption Study

### 3.1 Contact Time Effect

**FIGURE 4:**
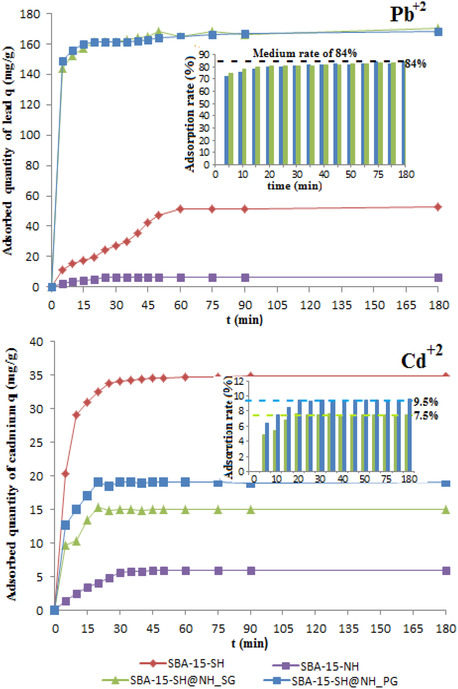
Kinetic studies of Pb^2+^ and Cd^2+^ adsorption by SBA‐15‐SH, SBA‐15‐NH, SBA‐15‐SH@NH_PG, and SBA‐15‐SH@NH_SG adsorbents at ambient temperature; *t* = 0–180 min; [Me]_0_ = 40mg/L; dose of adsorbent = 0.2 g/L and at pH solution (5.3 for lead and 6.1 for cadmium, respectively). Inset the adsorption rate evolution of Pb^2+^ and Cd^2+^ by only the bifunctionalized adsorbents.

Figure 4 illustrates the evolution of lead and cadmium adsorbed quantities by the mono‐ and bifunctionalized adsorbents as a function of time. Just for the contact time study, the monofunctionalized SBA‐15‐SH and SBA‐15‐NH are used as adsorbents to compare them with the bifunctionalized adsorbents and to follow the evolution of the adsorption phenomena.

Our results indicate clearly that the adsorption is time dependent and the process is moderately fast; the adsorbed quantities of lead and cadmium ions increase sharply within the first 10–15 min, then slowly; the equilibrium is reached after 50 min. The uptake capacities of lead ions by the prepared bifunctionalized materials were enhanced (164 and 168 mg/g for SBA‐15‐SH@NH_SG and SBA‐15‐SH@NH_PG, respectively) compared with the monofunctionalized thiolated (51 mg/g) or aminated ones (6 mg/g). In contrast, the cadmium uptake by the bifunctionalized materials (19 and 16 mg/g for SBA‐15‐SH@NH_SG and SBA‐15‐SH@NH_PG, respectively) appears between the thiolated and aminated (6 mg/g for both) load capacity, respectively. The two bifunctionalized materials both show similar adsorbed quantities of lead ions, while the slight difference in adsorption uptake of cadmium ions values can be due to the different incorporation rates of thiol and amine groups (as shown in Table 1).

According to Pearson's HSAB theory [26, 27], the high affinity between sulfur coordination sites (a soft base) and Pb^2+^ (a Lewis and soft acid) plays the main role in the adsorption process. Upon grafting of the amine groups, only after the complete saturation of the sulfur coordination sites, Pb^2+^ will adsorb to the amine groups that work as secondary sorption sites [32]. Accordingly, the retention capacities of Pb^+2^ will be further exalted. Practically, the introduction of amino groups has no effect on the adsorption of Cd^+2^.

Considering the high affinity of thiol for lead ions and comparing the adsorption capacities between mono‐ and bifunctionalized adsorbents, the additional presence of amine groups serves to make this coordination site more attractive to Pb^2+^ than to Cd^2+^, still based on HSAB theory.

This behavior conducts to the deduction that there is a synergic effect created after the incorporation of –SH and –NH_2_ fragments resulting, in a considerable increase of lead ion adsorption capacities against a remarkable decrease in those of cadmium ions.

In addition, from Table 1 there is no correlation between textural characteristics of the bifunctionalized adsorbents (their specific surface area) and their affinities to adsorb lead or cadmium ions. Hence, it is a chemical adsorption and not a physical one.

Thereafter, the contact time effect study was examined by analyzing the experimental data using pseudo‐first order (Equation (1)) [46] and pseudo‐second order (Equation (2)) [47]. These two models were expressed as following:

(1) $$Ln\;\left(q_{e}-\;q_{t}\right)\;=\;ln\;q_{e}-\;k_{1}t$$

(2) $$t/q_{t}\;=\;1/k_{2}{q_{e}}^{2}\;+\;t/q_{e}$$

where *q* *_e_* and *q* *_t_* (mg/g) are the adsorbed quantities at equilibrium and at *t* time, respectively; *k*_1_ (min^−1^) and *k*_2_ (min.g/mg) are the pseudo‐first‐order and pseudo‐second order constants, respectively; where *k*_1_ can be evaluated from the slope of the *Ln* (*q* *_e_*–*q* *_t_*) versus *t* and the plot of *t*/*q* *_t_* versus *t* yields the *q* *_e_* as slopes and *k*_2_ as intercepts, respectively.

**FIGURE 5:**
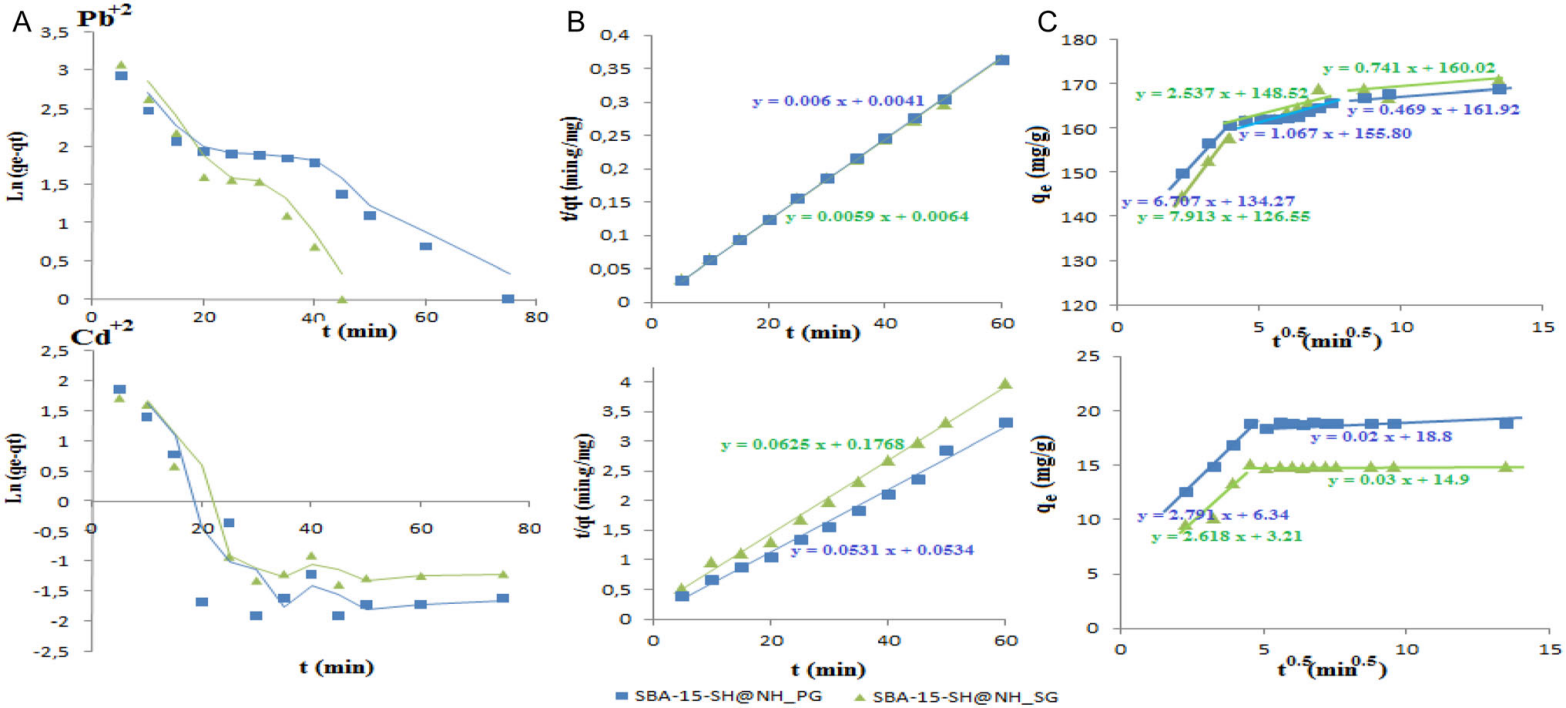
Kinetic adsorption of Pb^2+^ and Cd^2+^ onto SBA‐15‐SH@NH_SG and SBA‐15‐SH@NH_PG‐ Modeling by pseudo‐first (graphs A), pseudo‐second (graphs B) orders, and intraparticle diffusion models (graphs C).

The corresponding graphs are shown in Figure 5 and the different kinetic parameters with correlation coefficients (*R* ^2^) are listed in Table 2.

**TABLE 2:** Pseudo‐second‐order kinetic parameters.

Adsorbents	Metallic ions							
	Pb^2+^				Cd^2+^			
	*q* *_e_* a, mg/g	*q* *_e_* a, mg/g	10^3^ *k*_2_ b, min.g /mg	*R* ^2^ c	*q* *_e_* a, mg/g	*q* *_e_* a, mg/g	10^3^ *k*_2_ b, min.g /mg	*R* ^2^ c
SBA‐15‐SH@NH_SG	164	166.66	8.78	0.99	19	18.8	0.053	0.99
SBA‐15‐SH@NH_PG	168	169.5	5.44	0.99	16	16	0.022	0.99

a is the experimental and calculated adsorbed quantities (mg/g) of lead and cadmium ions, respectively.

b is the pseudo‐second‐order (min g/mg).

c is the correlation coefficien.

As seen in both Figure 5 and Table 2, there is a good agreement between the experimental and calculated adsorbed quantity values, which proves that the adsorption kinetics of lead and cadmium ions at room temperature using SBA‐15‐SH@NH_PG and SBA‐15‐SH@NH_SG as adsorbents is more consistent with the pseudo‐second‐order defined with a high correlation coefficient (= 0.99). This kinetic model suggests that the rate‐limiting step for both Pb(II) and Cd(II) adsorption is chemisorption involving valence forces through sharing or exchanging electrons between the adsorbent and adsorbate as covalent forces and ion exchange [48].

Generally, the adsorption mechanism can occur from three sequential steps: (i) external diffusion of the adsorbate through the boundary layer of the adsorbent surface, (ii) intraparticle diffusion, and (iii) adsorption of the adsorbate onto active sites of the adsorbent [49]. To have more details on the adsorption process, the intraparticle diffusion model [50, 51] defined by Equation (3) is also used:

(3) $$q_{t}=\;k_{i}t^{0.5}+\;C_{i}$$

where: *k*_i_ (mg g^−1^ t^−0.5^) is the intraparticle diffusion rate constant and *C* (mg/g) is an arbitrary constant related to the thickness of the boundary layer. *k* *_i_* and *C* *_i_* are calculated by the slope and the intercept of the straight‐line portion of plotting *q* *_t_* versus *t* ^0.5^. If the plot of *q* *_t_* versus *t* ^0.5^ gives line passing by the origin, i.e. *C* *_i_* = 0, the adsorption process will be controlled only by the intraparticle diffusion.

The results are shown also in Figure 5 (see graph C), and intraparticle diffusion model parameters are summarized in Table 3.

**TABLE 3:** Intraparticle diffusion model parameters.

Adsorbents	Parameters	Metallic ions				
		Pb^2+^			Cd^2+^	
		1^st^ step	2^nd^ step	3^rd^ step	1^st^ step	2^nd^ step
SBA‐15‐SH@NH_SG	*K*_i_, mg g^−1^ min^−0.5^	7.913	2.537	0.741	2.618	0.03
	Ci, mg/g	126.55	148.52	160.02	3.21	14.9
	R^2^	0.99	0.85	0.73	0.90	0.20
SBA‐15‐SH@NH_PG	*K*_i_, mg g^−1^ min^−0.5^	6.707	1.067	0.469	2.791	0.02
	Ci, mg/g	134.27	155.80	161.92	6.34	18.8
	*R* ^2^	0.99	0.82	0.87	0.99	0.10

From this latter, we can see that the diffusion processes of lead and cadmium ions are different from each other. The obtained multilinear plots suggest the existence of two or more steps in the process when *C* *_i_* ≠ 0 [52]. However, the lead ions diffusion occurs through three stages, while the cadmium ions' diffusion is contributed only through two stages. In all cases, each stage is characterized by its own rate constants (Table 3). In the lead ions part, the rate constants of the first step are higher during the first 20 min of adsorption; it means the fast out‐diffusion of lead ions at the external surface of the adsorbents. After that, the rate constants become lower during the following 25–30 min (2^nd^ step), which is linked with the spread of lead ions from the outer surface of matter to the interior; it corresponds to the internal diffusion step. At last, the active sites of adsorbents had progressively attained adsorption, saturation accompanied with a considerable decrease in rate constant values. These results agree well with those obtained by Liang et al. [53]. Although the adsorption of cadmium ions has in common only the external diffusion stage (the first step for 20 min) with moderate rate constants, the intraparticle diffusion is the rate‐limiting step of the ongoing adsorption stage [54].

Moreover, higher values of estimated diffusion rate constants indicate the fast reaching of lead ions of the external surface of the bifunctionalized adsorbents compared to cadmium ions, which aligns with the results of the pseudo‐second order model and shows clearly the difference between adsorption uptakes of these two ions.

### 3.2 Initial Metal Concentration Effect

The adsorption study of lead and cadmium ions from aqueous solution at room temperature using 0.2 g/L of bifunctionalized adsorbents as a function on initial metal concentration (from 10 to 100 mg/L) for 60 min and pH solution was investigated.

The obtained isotherms *q* *_e_* = *f* (*C* *_e_*) are presented in Figure 7 with their corresponding modeling according to Langmuir [55], Freundlich [56] and Temkin [57, 58] models. The nonlinear and linear forms are defined by the following Equations (4)–(6), respectively:

(4) $$q_{e}=\;q_{\max}b\;C_{e}/\left(1+bC_{e}\right);\;\;\;\;\;C_{e}/q_{e\;}\;=\;1/b\;q_{\max}+\;1\;/q_{\max}\;C_{e}$$

(5) $$q_{e}=\;K_{F}{C_{e}}^{1/n};\;\;\;\;\;Ln\;q_{e}=\;Ln\;K_{F}+\;1/n\;Ln\;C_{e}$$

(6) $$q_{e}=\;\left[RT/b_{T}\right]\;Ln\left(K_{T}.Ce\right);\;\;\;\;\;q_{e}=\;B_{T}Ln\;K_{T}+\;B_{T}LnC_{e}$$

where *C* *_e_* and *q* *_e_* are the concentration (mg/L) and the adsorption capacity (mg/g) of adsorbents at equilibrium, respectively; *b* is the constant of Langmuir isotherm (L.mg^−1^); *q*_max_ is the adsorption capacity at saturation (mg/g); *K* *_F_* and *n* are Freundlich parameters; *b* *_T_* is the variation of adsorption energy (kJ/mol), *K* *_T_* is the equilibrium binding constant (L/mg**),** *R* is the universal gas constant = 8.314 J/mol.K, *T* is the absolute temperature expressed in K and *B* *_T_* is Temkin constant related to the heat of adsorption (kJ/mol).

The Langmuir model is a theory that assumes monolayer coverage on identical and equivalent surface sites within the adsorbent. On the contrary, the Freundlich and Temkin isotherms are empirical models; the first one is specific for nonideal and reversible adsorption over a heterogeneous surface, but the second postulates a linear decrease of the heat of adsorption of all molecules in the layer with the surface coverage of adsorbent. It is a model that vividly describes the adsorption mechanism [58].

**FIGURE 6:**
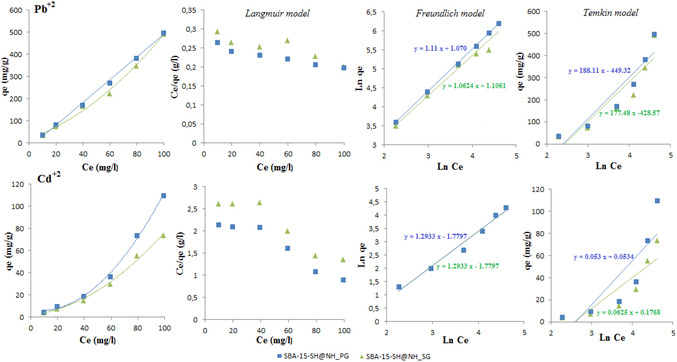
Adsorption, Langmuir, Freundlich, and Temkin isotherms of lead and cadmium ions adsorption study onto SBA‐15‐SH@NH_PG and SBA‐15‐SH@NH_SG‐Effect of initial metal concentration.

For both ions, the fitting of the adsorption isotherm illustrates the increase of adsorbed quantities with the initial metal concentrations (Figure 6). However, the isotherm is better presented and explained by the Freundlich model, with correlation coefficients varying between 0.97 and 0.99. In fact, this model is suitable for multilayer coverage with interaction among the adsorbed ions on a surface that is energetically heterogeneous. This obvious result was expected since the surface of the adsorbents contains two different types of adsorption sites, such as—SH and –NH_2_.

The values of Freundlich and Temkin parameters are listed in Table 4. Therefore, we conclude that the high adsorption quantities of lead ions against the low ones of cadmium ions are further confirmed by the high values of *K* *_F_* in the case of Pb(II) compared with those of Cd(II). In addition, *n* values are all less than 1, which means a chemical interaction between adsorbate molecules is established. By taking into consideration the heat of adsorption values determined by the Temkin model, the affinity of the used adsorbents via the adsorption of lead ions can be explained by the low values of *b* *_T_*, defined as the variation of adsorption energy against their considerable values in the case of cadmium ions. This result proves that the adsorption of lead ions is more favorable than the adsorption of cadmium ions.

**TABLE 4:** Freundlich and Temkin parameters.

Adsorbents	Metallic ions							
	Pb^2+^				Cd^2+^			
	Freundlich parameters		Temkin parameters		Freundlich parameters		Temkin parameters	
SBA‐15‐SH@NH_SG	*K*_F_	3.022	*B*_T_ a, kJ/mol	177,48	K_F_	0.168	*B*_T_ a kJ/mol	27.76
	*n*	0.94	*K*_T_ b, l/mg	0.089	*n*	0.77	*K*_T_ b l/mg	0.074
	*R* ^2^	0.97	*b*_T_ c, kJ/mol	13.91	*R* ^2^	0.98	*b*_T_ c kJ/mol	88.95
			*R* ^2^	0.83			*R* ^2^	0.77
SBA‐15‐SH@NH_PG	*K*_F_	2.915	*B*_T_ a, kJ/mol	188.11	*K*_F_	0.168	*B*_T_ a, kJ/mol	39.74
	*n*	0.9	*K*_T_ b, l/mg	0.091	n	0.77	*K*_T_ b, l/mg	0.078
	*R* ^2^	0.99	*b*_T_ c, kJ/mol	13.13	*R* ^2^	0.98	*b*_T_ c, kJ/mol	62.13
			*R* ^2^	0.88			*R* ^2^	0.72

a *B* *_T_* is determined by the fit of qe versus Ln Ce, *B*_T _= *R*.*T*/ Slope (*R* is the universal gas constant = 8.314 J/mol.K and *T* is the absolute temperature expressed in K).

b *K* *_T_* = exp(Intercept/Slope).

c *b* *_T_*  = R.T/B_T_.

### 3.3 Comparison of Results and Adsorption Mechanism Study

It is very important to compare the adsorption capacities of bifunctionalized adsorbents used in this work with similar synthesized ones reported in literature. However, there are few published articles that treat the simultaneous incorporation of thiol and amino groups onto the surface of SBA‐15 or analogous mesoporous materials.

We found two effective studies: one about using materials denoted as ATBS: amino and thiol‐bifunctionalized SBA‐15 [30] and another concerning adsorbents denoted as M–(N–S): amino and thiol‐bifunctionalized MCM‐41 [43]. The different results are recapitulated in Table 5.

**TABLE 5:** Adsorption capacities of Pb^2+^ and Cd^2+^ using thiol and amino bifunctional SBA‐15 or MCM‐41 adsorbents.

Adsorbent	Synthesis procedure		MPTMS and APTMS quantities	Adsorption experiment conditions	Adsorption uptake, mg/g Cd^2+^ Pb^2+^		References
ATBS: Amino and Thiol bifunctionalized SBA‐15	Bifunctionalized adsorbents 0.2 ATFS (cocondensation followed by grafting)		*V*_MPTMS_ = 5.13 mL *V*_APTMS_ = 3 mL	[Me^2+^]_0_ = 3 mM T = 25°C pH = 5.5 ± 0.05 *t* = 24 h dose_adsorbent_ = 1.0 g/L	**38**	**120**	[30]
	Monofunctionalized adsorbent 0.2 TFS (cocondensation)		*V*_MPTMS_ = 5.13 mL		23	19	
M–(N–S): Amine and Mercapto Bifunctionalized MCM‐41	Bifunctionalized adsorbents M–(N–S) (two successive prehydrolysis grafting of Amino and Mercapto)		*V*_MPTMS_:/ *V*_APTMS_:/	[Pb^2+^]_0_ = 0.5 m mol/L T = 25°C pH =/ *t* = 3h dose_adsorbent_ = /	—	**90.91**	[43]
SBA‐15‐SH@NH: Thiol and Amino Bifunctionalized SBA‐15	Bifunctionalized adsorbents SBA‐15‐SH@NH	Postgrafting: SBA‐15‐SH@NH_PG	*m*_MPTMS_ = 1g *m*_APTMS_ = 1 g	[Me^2+^]_0_ = 40 mg/L T = 25°C pH = 5.3 ± 0.1 (for Leadd) pH = 6.1 ± 0.1 (for cadmium) *t* = 24 h dose_adsorbent_ = 0.2 g/L	*19*	**168**	This work
		Successive grafting: SBA‐15‐SH@NH_SG			*16*	**164**	
	Monofunctionalized adsorbents	Grafting: SBA‐15‐SH	*m*_MPTMS_ = 1g		34	51	
		Grafting: SBA‐15‐NH	*m*_APTMS_ = 1g		6	6	

Despite the difference in the synthesis conditions of bifunctionalized materials as well as the conditions of adsorption experiments, we note that the adsorption quantities of lead ions are more significant and higher than those of monofunctional adsorbents. On the other hand, the postgrafting appears more efficient than the cocondensation in the preparation of bifuntionalized materials (our work).

As regards the adsorption mechanism involved, our results of the kinetic study suggest a chemisorption that mainly consists of covalent bonds, such as complexation reaction and ion exchange. So, the high values of lead adsorption quantities clearly demonstrate that the covalent bond adsorbate–adsorbent is so strong that ion exchange, which is commonly a reversible process [59]. In the case of cadmium ion adsorption, the reversibility of the process is dominant.

In parallel, the study of adsorption isotherms agrees with the Freundlich model involving a heterogeneous surface of bifunctionalized adsorbents corresponding to the existence of –SH and –NH as two different types of adsorption sites. Using X‐ray photoelectron spectroscopy, Tang et al. [30] were able to prove that S atoms of thiol groups become electronegative after sorption, and one Pb might react with two thiol groups to form a complex. Moreover, the formation of the covalent bond N—Pb also exists, whereas no peak is observed corresponding to the N—Cd bond. This may explain the origin of the synergic effect due mainly to the simultaneous presence of thiol and amino groups.

### 3.4 pH Effect and Reuse of Adsorbents

Due to the presence of different functional groups on the surface of the adsorbent, pH values play an important role, particularly in metal ions from aqueous solution [60, 61, 62]. Also, pH influences the pollutant species in solution by controlling their electrostatic interactions. In our experiments, the adsorption evolution of Pb(II) and Cd(II) at ambient temperature was achieved under the following conditions: [Me]_0_ = 40 mg/L; adsorbent dose = 0.2 g/L and *t* = 60 min. The pH was varied between 2 and 10 using HCl (0.2 M) or NH_4_OH (0.5 M).

**FIGURE 7:**
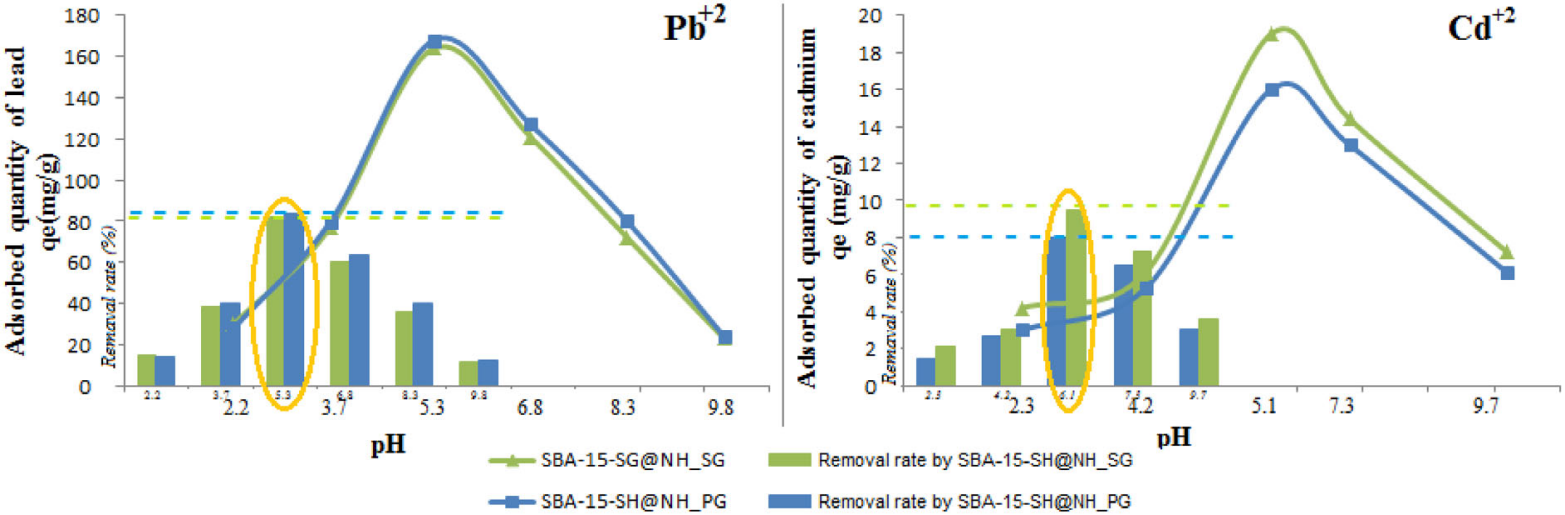
Adsorption and rate removal (inset) evolutions of Pb^+2^ and Cd^+2^ onto SBA‐15‐SH@NH_SG and SBA‐15‐SH@NH_PG‐effect of solution pH.

The results are shown in Figure 7. As observed, the adsorption of Pb(II) and Cd(II) ions by SBA‐15‐SH@NH_SG and SBA‐15‐SH@NH_PG reaches its maximum at pH values of 5.3 and 6.1, respectively, which are their pH solutions. However, the removal rate of lead ions is approximately 84% and less than 10% for cadmium ions. At an acidic pH, lower amounts adsorbed are obtained for cadmium ions, but we are still witnessing notable lead ions adsorption capacities (removal % varied from 15 to 40% while pH value varied from 2 to 4). The lone pair electrons in free thiol –SH and amino –NH_2_ groups grafted onto the surface of SBA‐15 in the presence of the H_3_O^+^ ions became protonated. As a result, their presence generates a strong electrostatic repulsion between the different positive charges, including those of lead and cadmium ions [63]. When pH values increased, protons deprotonated from functional groups, making the surface of both adsorbents more negatively charged. This increases Pb^2+^ and Cd^2+^ retention until reaching their maximum at pH 5.3 and 6.1. In contrast, at alkaline pH (>8), the precipitation of metals to their hydroxide forms, such as MeOH^+^, Me(OH)^2^, Me(OH)^−^_3_, and Me(OH)_4_, is favored and the availability of adsorbents diminishes. From pH 6 to pH 8, the rate of removal of cadmium ions is still constant due to the predominance of Cd(II) in the form of Cd(OH)^+^ [62, 64]. In the case of lead ions, the rate of removal decreases slowly, passing from 63% to 40%.

**FIGURE 8:**
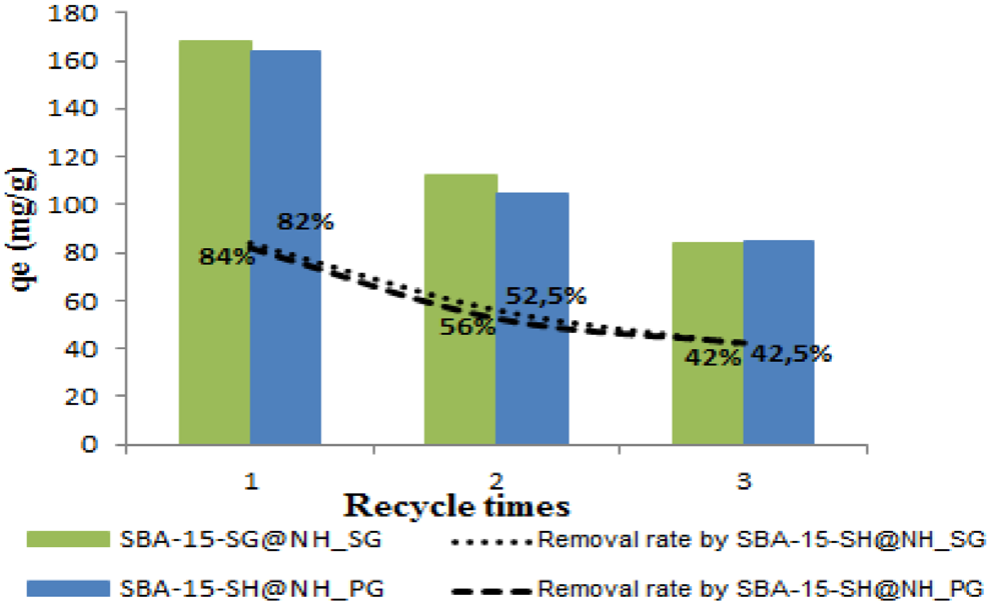
Adsorption of Pb^+2^ onto SBA‐15‐SH@NH_SG and SBA‐15‐SH@NH_PG at ambient temperature, [Pb]_0_ = 40 mg/L and *t* = 60 min. Reuse study.

The adsorbent reuse test was carried out by treating them with HCl (1 M) to desorb the metal ions for 1 h at room temperature. The solid was filtered and dried in ambient air. The recovered solid was reused for lead and cadmium ions adsorption as described above. This operation was repeated three times. As indicated in Figure 8, the adsorption efficiency of both adsorbents, SBA‐15‐SH@NH_SG and SBA‐15‐SH@NH_PG, appears maintained even after three regenerative cycles, but the adsorption quantities decrease from 168–164 to 84–85 mg/g. Practically, no uptake capacity is shown in the case of cadmium ion adsorption. The efficiency of reuse of adsorbents proves their resistance to acid treatment and the persistence of the synergic effect coming from the existence of S and N atoms onto the surface of the used adsorbents.

## 4 Conclusion

The simultaneous introduction of thiol and amino functions onto the surface of mesoporous material SBA‐15 was successfully achieved by post and SG. However, the textural properties of the resulting materials were significantly reduced. Furthermore, the adsorption capacities of lead and cadmium ions using both adsorbents show a synergic effect. In the case of lead ions, the adsorbed amounts were elevated compared to the monofunctionalized material with thiol groups (from 50 to 164–168 mg/g) when the adsorption of cadmium ions was reduced (from 35 to 16–19 mg/g). At this time, this difference can be explained by the fact that these two adsorbents have different profiles toward lead and cadmium ions. Moreover, we reported during the kinetic study that the diffusion of ions occurs through three stages in the case of lead ions, and consequently, significant adsorbed quantities are revealed, whereas there were only two stages for cadmium ions.

We can conclude that the presence of these two functions not only changes the complexation site natures of adsorbents but also generates a significant selectivity between lead and cadmium ions. It seems very important to use these adsorbents in the treatment of wastewater, industrial wastewater management and soils loaded with lead ions and especially in multimetallic systems.

## Supporting Information

Additional supporting information can be found online in the Supporting Information section.

## Conflicts of Interest

The authors declare no conflicts of interest.

## Supporting information

Supplementary Material

## Data Availability

The data that support the findings of this study are available on request from the corresponding author. The data are not publicly available due to privacy or ethical restrictions.
