# Supplementary material for: Hybrid Mesoporous Bifunctional Adsorbents of PbII and CdII Ions from Aqueous Solution at Ambient Temperature
Source: ChemistryOpen. 2026 Apr 28;15(5):e202500565. doi: 10.1002/open.202500565 (PMC13125856; doi:10.1002/open.202500565)
Supplement: Supplementary file 1 — Supplementary Material [file OPEN-15-e202500565-s001.pdf]

Supporting Information

Chemicals and materials

Pluronic P123 (Aldrich, 80%), tetraethyl orthosilicate (TEOS) (Aldrich), 3-mercaptopropyltriethoxysilane (MPTES) (Aldrich, 95%), 3-aminopropyltrimethoxysilane (APTMS) (Aldrich, 97%), hydrochloric acid (Riedel-de Haën, 32%), ammonium hydroxide (Sigma-Aldrich), lead nitrate (Aldrich, 99%), cadmium nitrate (Aldrich, 99%), toluene (Sigma-Aldrich, 99.9%), ethanol (Riedel-de Haën, 96%) were used as received.

Preparation method

After the synthesis of the mesoporous silica material SBA-15 as mentioned in the literature [28] by using the triblock copolymer poly (ethylene oxide)-poly (propylene oxide)-poly (ethylene oxide) (P123) as a structure directing agent and tetra-ethyl-orthosilicete (TEOS) as a silica source in acidic media ([HCl]=0.24M), the mono- and bi-functionalization of SBA-15 by 3-mercaptopropyltrimethoxysilane (MPTMS) and/or 3-aminopropyltrimethoxysilane (APTMS) were achieved as follows [29]: 1g of SBA-15 calcined and dried under vacuum at 110°C was introduced into 25 ml of toluene containing MPTMS, APTMS or MPTMS+APTMS (see scheme 1). The mixture is stirred for 8 hours at reflux. Then, the recovered solid is filtered and washed with toluene and ethanol, successively. Thereafter, the solid is extracted with ethanol for 8 hours again. The solid is then dried at room temperature. The resulting materials are denoted as: thiolated material SBA-15-SH, aminated material SBA-15-NH, bi-functionalized materials SBA-15-SH@NH_PG and SBA-15-SH@NH_SG.
The grafting of these organic functions was accompanied by the characterization of the obtained materials by different methods such as XRD, N_2 adsorption-desorption, and FT-IR analyses. In parallel, the performance of these adsorbents was shown by the study of adsorption of lead and cadmium ions from aqueous solution as function of synthesis procedure, contact time, initial metallic concentration, pH, and reuse of adsorbent at ambient temperature.

Adsorption experiments

Metal ion adsorption on SBA-15-SH, SBA-15-NH, SBA-15-SH@NH_SG and SBA-15-SH@NG_PG were carried out in batch experiments at 25±1°C at solution pH where Pb (II) and Cd (II) were detected. A 0.2 g/l as adsorbent dose was thoroughly mixed into 25 ml aqueous solution containing selected concentration of metal ion. The data used for the adsorption isotherms were obtained using various concentrations (5-100 mg/l) and 180 min of contact time.
The metal ion uptake capacity and the corresponding adsorption rate were calculated by analyzing the metal concentration of the solution before and after adsorption using the following equation:

$$q_{ads} = \frac{(C_0 - C_t) \,.\, V}{m} \quad \text{and} \quad \text{Removal (\%)} = \frac{(C_0 - C_t) \,.\, 100}{C_0}$$

where:
C_0 and C_t: the initial and final concentrations of the metal ions in solution (mg/L), respectively; V: volume of the metal solution (L); m: dry weight of the adsorbent (mg).

Characterization methods

The structural evolution of resulted materials was followed by X-ray diffraction. XRD spectra were performed on a D5000 Siemens powder diffractomer (Cu Kα) in the angle range of 0.5-2° 2Θ with the steps of 0.01°. Specific surface area was calculated from adsorption isotherms by the BET (Brauauer-Emmett-Teller) method; while the pore volume and mean pore size were obtained using adsorption isotherms by BJH (Barett-Joyner-Halenda) method using a Micromeritics ASAP2010 at liquid N_2 temperature (-190°C). The functional groups were analyzed by FT-IR which infrared spectrum were acquired from KBr pellets in the 4000-400 cm^{-1} range using a Perkin Elmer FT-IR spectrophotometer.
The determination of lead and cadmium concentration (mg/L) in the filtrate after adsorption experiments was achieved using an atomic absorption spectrometer WFX-130 model equipped with Varian multi-element hollow cathode lamps and an air-acetylene burner. The wavelengths (nm) selected for the detection were 283.3 nm for lead ions and 228.8 nm for cadmium ions.
